# High-fidelity detection, subtyping, and localization of five skin neoplasms using supervised and semi-supervised learning

**DOI:** 10.1016/j.jpi.2022.100159

**Published:** 2022-11-26

**Authors:** James Requa, Tuatini Godard, Rajni Mandal, Bonnie Balzer, Darren Whittemore, Eva George, Frenalyn Barcelona, Chalette Lambert, Jonathan Lee, Allison Lambert, April Larson, Gregory Osmond

**Affiliations:** a Pathology Watch, 497 West 4800 South, Suite 201, Murray, UT 84123, USA; b Cedars-Sinai Medical Center, 8700 Beverly Blvd, Los Angeles, CA 90048, USA; c Kirk Kerkorian School of Medicine at UNLV, University of Nevada, Las Vegas, Mail Stop: 3070, 2040 W Charleston Blvd., Las Vegas, NV 89102-2244, USA; d Bethesda Dermatopathology Laboratory, 1730 Elton Road, Silver Spring, MD 20903, USA; e Intermountain Healthcare, Saint George Regional Hospital, Department of Pathology, 1380 East Medical Center Drive, Saint George, Utah 84790, USA

**Keywords:** Artificial intelligence, Supervised learning, Semi-supervised learning, Skin cancer detection, Whole-slide imaging, Dermatopathology

## Abstract

**Background:**

Skin cancers are the most common malignancies diagnosed worldwide. While the early detection and treatment of pre-cancerous and cancerous skin lesions can dramatically improve outcomes, factors such as a global shortage of pathologists, increased workloads, and high rates of diagnostic discordance underscore the need for techniques that improve pathology workflows. Although AI models are now being used to classify lesions from whole slide images (WSIs), diagnostic performance rarely surpasses that of expert pathologists.

**Objectives:**

The objective of the present study was to create an AI model to detect and classify skin lesions with a higher degree of sensitivity than previously demonstrated, with potential to match and eventually surpass expert pathologists to improve clinical workflows.

**Methods:**

We combined supervised learning (SL) with semi-supervised learning (SSL) to produce an end-to-end multi-level skin detection system that not only detects 5 main types of skin lesions with high sensitivity and specificity, but also subtypes, localizes, and provides margin status to evaluate the proximity of the lesion to non-epidermal margins. The Supervised Training Subset consisted of 2188 random WSIs collected by the PathologyWatch (PW) laboratory between 2013 and 2018, while the Weakly Supervised Subset consisted of 5161 WSIs from daily case specimens. The Validation Set consisted of 250 curated daily case WSIs obtained from the PW tissue archives and included 50 “mimickers”. The Testing Set (3821 WSIs) was composed of non-curated daily case specimens collected from July 20, 2021 to August 20, 2021 from PW laboratories.

**Results:**

The performance characteristics of our AI model (i.e., Mihm) were assessed retrospectively by running the Testing Set through the Mihm Evaluation Pipeline. Our results show that the sensitivity of Mihm in classifying melanocytic lesions, basal cell carcinoma, and atypical squamous lesions, verruca vulgaris, and seborrheic keratosis was 98.91% (95% CI: 98.27%, 99.55%), 97.24% (95% CI: 96.15%, 98.33%), 95.26% (95% CI: 93.79%, 96.73%), 93.50% (95% CI: 89.14%, 97.86%), and 86.91% (95% CI: 82.13%, 91.69%), respectively. Additionally, our multi-level (i.e., patch-level, ROI-level, and WSI-level) detection algorithm includes a qualitative feature that subtypes lesions, an AI overlay in the front-end digital display that localizes diagnostic ROIs, and reports on margin status by detecting overlap between lesions and non-epidermal tissue margins.

**Conclusions:**

Our AI model, developed in collaboration with dermatopathologists, detects 5 skin lesion types with higher sensitivity than previously published AI models, and provides end users with information such as subtyping, localization, and margin status in a front-end digital display. Our end-to-end system has the potential to improve pathology workflows by increasing diagnostic accuracy, expediting the course of patient care, and ultimately improving patient outcomes.

## Highlights

• Including a large dataset of hand-drawn annotations increases the sensitivity of AI.
• Integrated supervised and semi-supervised learning is superior to other AI models.
• Multi-level training permits subtyping, localization, and margin detection.
• Dermpath involvement in model development can ultimately improve model performance.
• Increased confidence in AI-assisted diagnoses could lead to improved patient care.

## Graphical abstract

**Figure:**
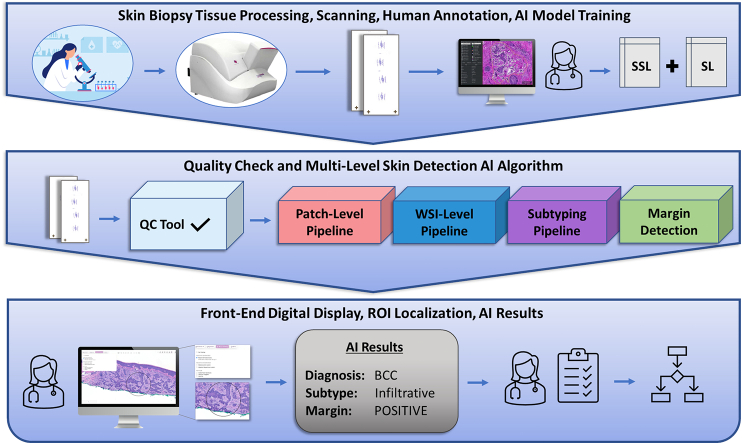

## Introduction

Skin cancers—the most common malignancies diagnosed worldwide—account for over 30% of all cancer diagnoses.^1^, ^2^, ^3^ Factors such as a growing and aging global population and increased solar UV radiation have resulted in a substantial increase in the incidence of skin cancer in recent years,^4^, ^5^, ^6^ which is projected to continue rising in the decades to come. The incidence of non-melanoma skin cancers (NMSCs)—which cause 5400 deaths globally each month^4^—has increased by more than 77% over the last several decades,^7^ while the prevalence of melanoma has increased by more than 250% since 1973 in children, adolescents, and young adults.^8^ Although the vast majority of skin cancers (99%) are classified as (NMSCs),^9^^,^^10^ more than 75% of deaths from skin cancers are attributed to melanoma.^3^^,^^11^^,^^12^

As with any malignancy, the early detection and treatment of pre-cancerous and cancerous skin lesions significantly improves survival rates, most notably in cases of melanoma.^3^^,^^13^, ^14^, ^15^ When diagnosed and treated locally, melanoma has a 99% five-year survival rate; however, after spreading to distant organs, tissues, or lymph nodes, the 5-year survival rate plummets to 30%.^13^ Despite the importance of early detection for long-term survival, a shortage of pathologists, increased workloads, and the complexity of histopathological diagnoses contribute to high rates of diagnostic discordance in a broad range of cancers, even among experienced pathologists.^16^, ^17^, ^18^, ^19^, ^20^, ^21^ Skin lesions are no exception, as is evidenced by high interobserver discordance rates among dermatopathologists in categorizing lesions as malignant vs nonmalignant,^22^, ^23^, ^24^, ^25^, ^26^, ^27^ where consensus rates for melanocytic lesions can be as low as 25%.^21^^,^^23^ Such high discordance rates could be a result of the fine-grained variability in morphology, numerous benign histologic mimickers of malignant skin neoplasms, and inherently subjective interpretation of such visual data.^20^^,^^28^^,^^29^ Moreover, it has been demonstrated that a dermatopathologist’s tendency towards rendering a malignant as opposed to non-malignant (i.e., atypical) diagnosis is subject to diagnostic drift favoring the former over time.^30^ Thus, although the human microscopic evaluation of hematoxylin and eosin stained tissue sections has been the cornerstone of histological cancer diagnoses for the past century,^25^^,^^31^^,^^32^ the need for more objective and standardized methods of histochemical detection and classification that meet the current demand is of paramount clinical concern.^23^^,^^31^

Digital pathology—the practice of using high-resolution digital imaging to manage and analyze data from digitized specimen slides—is rapidly becoming the standard of care in the field of pathology.^33^ Concomitantly, substantial advancements in artificial intelligence (AI) algorithms and data management capabilities have catapulted the marriage of digital pathology and AI into the forefront of modern clinical practice.^33^^,^^34^ The integration of digitized whole slide images (WSIs), advanced image processing technologies, and advanced machine learning (ML) models into pathology workflows has the potential to revolutionize clinical oncology by improving efficiency, diagnostic accuracy, and ultimately outcomes.^31^^,^^33^^,^^35^^,^^36^ In recent years, complex ML models, such as deep learning with convolutional neural networks (CNNs), have been used to detect and classify malignant cells and tissues in a wide variety of cancers (e.g., bladder, lungs, colorectal, lymph node, breast, brain, and prostate^19^^,^^31^^,^^35^^,^^37^, ^38^, ^39^, ^40^, ^41^, ^42^, ^43^), as well as cancers of the skin.^20^^,^^21^^,^^28^^,^^44^^,^^45^

However, while AI models generally perform well in binary histopathological comparisons (i.e., malignant vs. benign), many AI models that rely solely on semi-supervised (SSL) or weakly supervised learning do not outperform or only slightly outperform human pathologists when faced with classifying the pathobiological spectrum of neoplasia encountered in routine specimens.^19^, ^20^, ^21^^,^^45^, ^46^, ^47^, ^48^ As a result, the sensitivity or accuracy of existing AI models in classifying melanocytic lesions and forms of skin cancer, wherein greatly nuanced pathological gradation exists, rarely exceeds 95%.^20^^,^^44^^,^^49^, ^50^, ^51^ A recent study that used weakly supervised multiple-instance learning (MIL) to perform a hierarchical classification of a WSI dataset that was enriched for rare melanocytic specimens only achieved sensitivities of 83% and 85% for high and intermediate risk classes in the “melanocytic suspect class”, respectively.^44^ Another study that used a weakly supervised CNN (i.e., slide-level diagnoses) to decipher between melanomas and benign nevi only achieved a concordance rate of 81% between human pathologists and the AI algorithm.^21^ Furthermore, AI models trained using only semi- or weakly supervised learning tend to underperform when analyzing fine-grained characteristics such as grading and subtyping, or distinguishing between different classes of malignant lesions,^19^^,^^44^^,^^52^ resulting in a reliance on coarse-grained, slide-level diagnoses.^46^^,^^53^ Therefore, to improve patient care and drive the adoption of AI models in clinical workflows, algorithm development should be optimized to effectively interpret the fine-grained characteristics and accurately classify the spectrum of diagnostically challenging lesions in dermatopathology.

Supervised learning (SL) algorithms use training datasets that rely on ground-truth labels provided by human annotations of input variables (i.e., features) to predict the corresponding outputs, allowing SL models to emulate the expert annotator in predicting features of unknown inputs.^54^ Just as deep neural networks have become the gold-standard for image segmentation and classification,^54^^,^^55^ the SL approach could be considered the gold-standard for training AI algorithms that are designed to predict features in biomedical imaging.^56^, ^57^, ^58^ Likewise, SL algorithms trained on the pixel-level delineation of features annotated by expert dermatopathologists could enable tasks such as the localization of malignant cells and tissues within each slide, non-binary classifications such as subtyping, and margin detection with exceptionally high accuracy.^59^ However, although fine-grained training data labels convey clear benefits in terms of localization, accuracy, and sensitivity in comparison to coarse-grained algorithms, SL is not standard practice in digital dermatopathology. The few studies that have reported on the use of SL in dermatopathology performed only binary classifications and are largely based on dermoscopy and microscopic ocular images ^60^^,^^61^, with only 2 studies featuring AI models trained on a small number of WSIs (i.e., 125–200).^49^^,^^59^ Although the importance of labeled training data for accurate and clinically useful AI model predictions is widely recognized, SL is unequivocally considered to be too tedious, time-consuming, and cost-prohibitive owing to the necessity of hand-drawn expert annotations and the high computing power required to store and process WSIs.^26^^,^^44^^,^^47^^,^^53^^,^^62^ Therefore, robust AI models that incorporate SL to perform highly accurate, fine-grained diagnoses of skin lesions are lacking.

**Fig.1:**
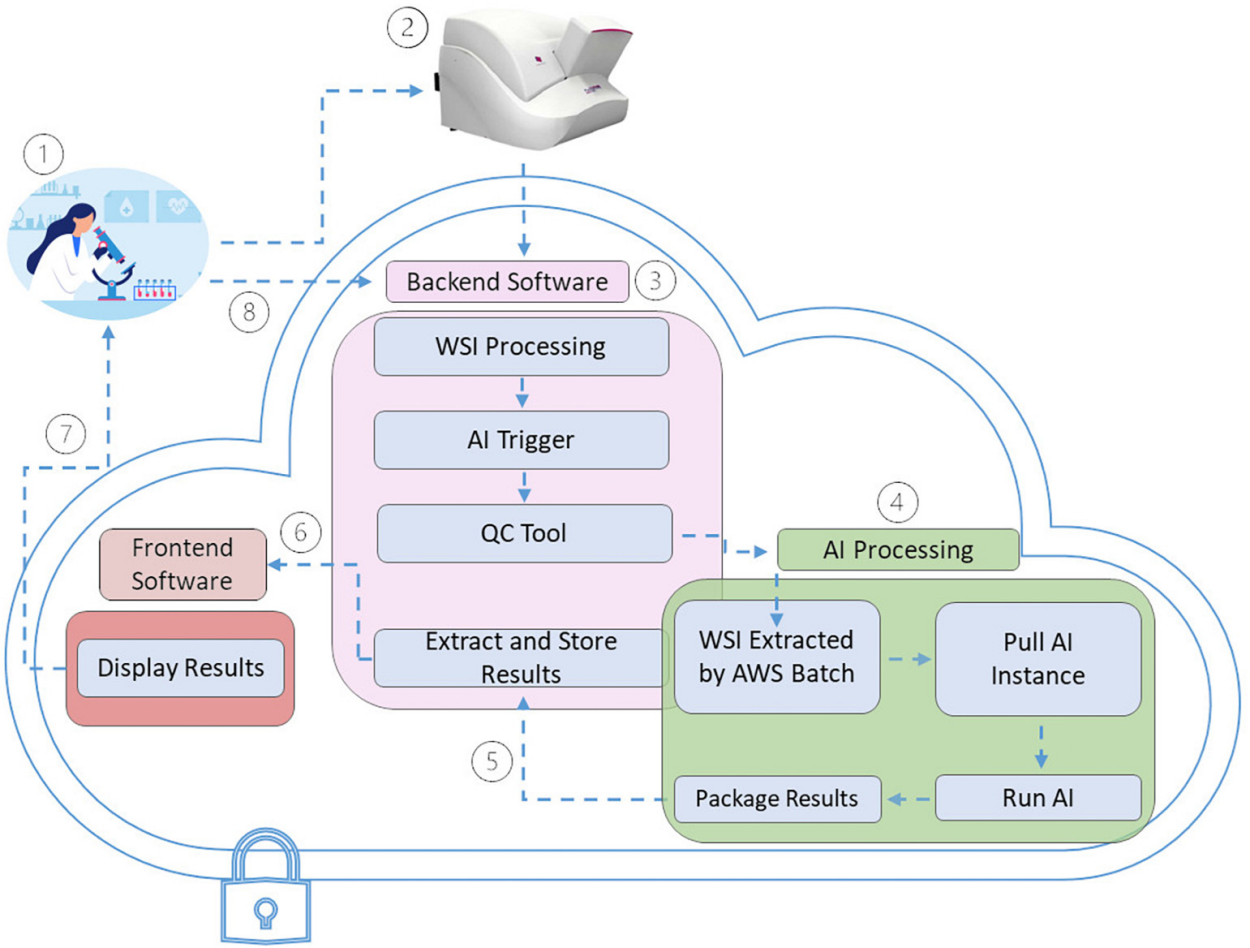
Graphical representation of closed-loop dermatopathology system developed by Pathology Watch (PW). Backend, frontend, and AI processing take place in the protected, enclosed environment of the Amazon Web Services (AWS) S3 cloud. (1) For retrospective analyses, the end-to-end system begins in the PW Laboratory (PWL), where formalin-fixed (FF) tissue samples were paraffin embedded (PE), stained with hematoxylin and eosin, sectioned, mounted on slides, and coverslipped according to PWL standardized protocols. The diagnosis is validated by a dermatopathologist. (2) Slides are subsequently scanned using a 3DHISTECH digital slide scanner. (3) Whole slide images (WSIs) are then picked up by AWS S3 and used by the backend for processing, including storing metadata in the database to make them available for the frontend and AI. The previous tool triggers the AI to pull the WSI, after which the QC tool checks slide quality prior to AI processing, where WSIs that do not pass QC checks are moved to the manual team for confirmation and resolution. (4) In AI processing, the AWS Batch service receives the classification trigger from the backend with metadata. AWS pulls the AI program from the elastic container registry and passes it to an elastic compute cloud (EC2) instance. A new EC2 instance is started and runs the AI program with the WSI. Results are then packaged and (5) extracted and stored in the database for later use by the backend software. (6) Results are requested from the backend software and displayed to the dermatopathologist to review the AI classification. (7) Dermatopathologist views the case and provides feedback on AI run cases, dermatopathologist feedback is compared to the AI prediction, and the results feed back into the AI development pipeline as data for semi-supervised learning.

To address the pressing need for AI models that include a large SL dataset to accurately classify both potentially malignant and benign lesions of the skin, we developed the computational pathology algorithm Mihm Diagnostic Assist (Mihm), named with permission after one of the founding fathers of dermatopathology, the late Martin Mihm.^63^ Mihm is the cornerstone of an end-to-end dermatopathology tool that comprises a dedicated lab for standardized tissue processing, dermatopathologists for expert annotations and sample acquisition, and back- and front-end software, the latter of which includes an interactive user interface that displays predictions of algorithmic outputs in natural language and regions of interest (Fig. 1). Mihm was trained on both a large-scale training dataset composed of 2000 WSIs with fine-grained, hand-drawn annotations for SL ground truth labels and >5000 WSIs in the weakly supervised dataset. To the best of our knowledge, Mihm is the first diagnostic pathology algorithm that incorporates a large-scale SL dataset to detect, classify, subtype, and localizing skin lesions on digitized WSIs.

## Methods

### Patient samples

The research protocol of the present study was reviewed and approved by an external institutional review board (IRB) and was determined to be exempt from regulations as defined under 45 CFR 46.104. For the purposes of the present study, all patient samples are deidentified upon digitization of glass slides using a whole slide imaging (WSI) system and assigned a unique file name that has no association with patient health information.

Formalin-fixed (FF) or formalin-fixed and paraffin-embedded (FFPE) tissue samples were previously acquired from patients during normal patient care and were processed into tissue blocks and glass histologic slides as described under “Slide preparation” below. Additionally, samples were received both previously stained with hematoxylin and eosin (H&E), as well as unstained, which were subsequently stained with H&E as described under “Slide preparation” below. All sample types were included in the training datasets, while testing and validation datasets consisted of only FF clinical samples that were received by Pathology Watch Laboratories (PWL) as FF clinical specimens and processed using PWL standardized protocols. For samples that were received as FF and FFPE tissues, on or after January 21, 2022, the margins of all biopsies and unoriented specimens were inked with a green tissue marking dye (Cat No. 0727-3, Cancer Diagnostics, Inc., Durham, NC, USA) on all but the epidermal surfaces during tissue grossing to automate margin detection (see “Margin detection: Overview”).

### Slide preparation

Inclusion criteria for skin specimens used in the present study were as follows: Source: skin; site: head/neck, trunk, extremities; clinical indication: lesion (e.g., bump, lump, tumor); patient age: ≥20 years old; stain type: H&E. The exclusion criteria can be seen in Table S1. Samples that had been previously prepared and archived as glass slides were scanned and deidentified. For instances in which additional samples of glass slides were created from archived tissue blocks, FFPE tissue was sectioned (5 μm), placed on standard 75 × 25 mm glass microscope slides and stained using a Gemini™ AS Automated Slide Stainer (Epredia; Portsmouth, NH, USA) according to standard protocols.

### WSI processing

Skin specimen WSIs that met the inclusion criteria were stained as previously described, digitized using 1 of 3 models of 3D HISTECH digital slide scanners (P100, P250, or P1000; 3D HISTECH, Budapest, Hungary). As images are normalized per pixel and data augmentation applied to offset variation in color values, variation in scanning devices only serves to diversify the *Training Set* and strengthen the *Validation Set*. The resulting .mrxs files were annotated by human annotators using the open-source pathology and bioimage analysis software QuPath (v0.1.3.5)^64^ to create ground-truth labels for SL (See “Datasets: training set”), as well as in other downstream processes.

### Overview of Mihm Diagnostic Assist

Mihm Diagnostic Assist (hereafter Mihm) is a computational pathology software that was developed as a novel method for the ML-based detection and localization of skin lesions from WSIs using neural networks. Mihm’s multi-stage, fully automated process includes the following consecutive steps: (i) quality assessment via Mihm’s automated QC tool (See “Mihm's automated QC tool”); (ii) skin lesion detection, localization, subtyping, and margin status via a series of specialized multi-scale CNNs and attention-based multiple instance learning (MIL; See “Mihm: Evaluation pipeline”); and (iii) the presentation of findings via a web-based front-end digital display (See “Continual learning: PW digital display, dermatopathologist feedback, and AI exporter”).

### Mihm’s automated QC tool

After digitization, Mihm analyzed WSIs for potential QC issues (e.g., incomplete scans, blurry regions, obstructed folded tissue, and artifacts) prior to Mihm's multi-level analysis for skin lesion detection, subtyping, and margin status. Only WSIs that passed the QC check proceeded to Mihm's subsequent stage of analysis. WSIs that were flagged by the automated QC tool for QC issues were reviewed by a QC/QA team and the WSI rescanned or re-processed if deemed appropriate.

#### QC training and validation dataset

To prepare the dataset to train the automated QC tool, we collected WSIs that were confirmed to have QC issues via manual review by QC/QA technicians along with WSIs which were confirmed to be of acceptable quality. In total, the *QC Training Dataset* consisted of 2000 WSIs with QC issues and 2000 WSIs without QC issues. For validation, we set aside 20% of the training data to assess the trained model.

#### QC dataset preparation and model training: ROI-level

For all WSIs, we used a segmentation model (U-Net^55^) to remove background/non-tissue areas and extract all tissue sections (i.e., one image per tissue section). For WSIs of unacceptable quality, the QC team assigned a QC label of blurry, folding, artifact, or incomplete to each tissue section of the WSI. For each tissue section image, we further generated a set of features, including variance of Laplacian (to detect blur), scan coverage (to detect incomplete regions), and pixel values (i.e., mean and standard intensity [0–255], brightness, luminosity, and saturation). Once fully prepared, the *QC ROI-level Training Dataset* consisted of images of tissue sections from all WSIs in the training set, along with the extracted features for each with each tissue section being assigned a QC label.

For the QC Model, we used a hybrid approach of a Random Forest (RF) and used the CNN model architecture ConvNeXt.^65^ The RF was fit to the tabular features of each tissue section image and the CNN was trained on the image with QC label pairs.

#### QC model analysis: WSI-level

For the QC tool to provide a WSI-level result, first, a prediction was generated for each of the QC issues for each tissue section in the WSI and then the ratio of poor-quality tissue sections to total sections was calculated. If >25% of tissue sections were identified as having QC issues, the WSI was flagged for QC issues. Otherwise, WSIs were determined to be of sufficient quality for further evaluation. Mihm's trained automated QC tool achieved 96% specificity and 94% sensitivity on the *Validation Set*.

### Datasets

*Training* (*Supervised* and *Weakly Supervised Subsets*), *Testing*, and *Validation Sets* were prepared to train and validate Mihm’s skin lesion detection algorithms. All annotations for all datasets were independently validated by a minimum of 2 board-certified dermatopathologists who concurred on the diagnosis and placement of annotations. The WSI counts for all sets are referenced in Table 1.

**Table 1:** Mihm development training and testing sets for predefined lesion classes.

	Training subset WSIs		Testing set WSIs
	SSL	SL	SSL
Melanocytic lesions	1268	632	621
Atypical squamous lesion	1286	507	720
Basal cell carcinoma	796	296	498
Seborrheic keratosis	316	94	141
Verruca vulgaris	173	78	107
Other	1322	581	1734
Total WSIs	5161	2188	3821

WSI: whole slide image, SSL: semi-supervised learning subset (i.e., weakly supervised subset), SL: supervised learning subset

#### Datasets: Training set

**Fig.2:**
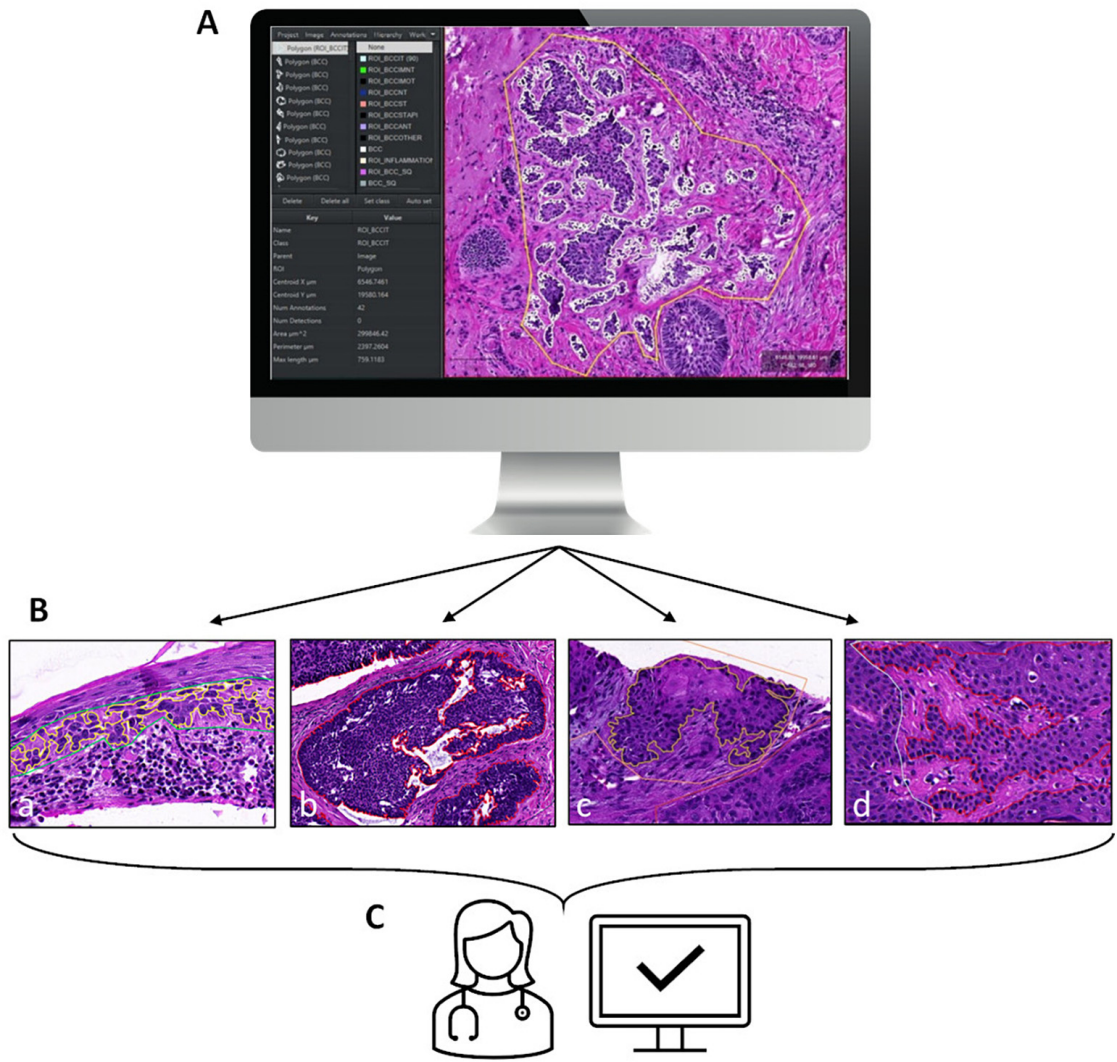
Supervised training hand-drawn annotation workflow. (A) Region of interests (ROIs) are selected in QuPath (v0.1.3.5) by a board-certified dermatopathologist (yellow outline) in the *Supervised Training Subset* during AI development prior to fine-grain selection by contractor. (B) Fine-grained ROI annotations (inner lines) are then performed by trained and supervised contractors after ROI selection by the first dermatopathologist annotator (outer lines). (a) Dermatopathologist selection: green outline, contractor selection: yellow outline; sample: actinic keratosis. (b) Dermatopathologist selection: blue outline, contractor selection: red outline; sample: basal cell carcinoma. (c) Dermatopathologist selection: pink outline, contractor selection: yellow outline; sample: squamous cell carcinoma. (d) Dermatopathologist selection: white outline, contractor selection: red outline; sample: seborrheic keratosis. All hand-drawn annotations were performed in QuPath. (C) The edges and diagnostic labels of cut ROIs are independently confirmed by another board-certified dermatopathologist to finalize the annotation

Mihm was first trained on the *Supervised Training Subset*, which consisted of 2188 WSIs from random de-identified samples collected between 2013 and 2018, with fine-grained mask annotations performed in QuPath at up to 40X magnification by a team of board-certified dermatopathologists for a wide range of commonly encountered skin lesions, including melanocytic lesions (e.g., melanoma, melanoma-in-situ, and a full spectrum of nevi), atypical squamous lesions (ASL) (i.e., squamous cell carcinoma [SCC], squamous cell carcinoma in-situ [SCCIS], actinic keratosis [AK]), basal cell carcinoma (BCC) (i.e., nodular, infiltrative, superficial, and mixed with subtypes), seborrheic keratosis (SK), verruca vulgaris (VV), as well as benign or healthy tissue. The workflow for performing supervised annotations for each WSI was as follows: (i) ROIs were first selected and labeled by a board-certified dermatopathologist (Fig. 2A); (ii) fine-grained cellular-level annotations (i.e., “nests”) were cut out by a pathologist-trained contractor from within the selected ROIs (Fig. 2B); (iii) the edges of cut ROIs and diagnostic labels were independently confirmed by another board-certified dermatopathologist to finalize the annotation (Fig.2C). When diagnostic or annotation discrepancies were found between 2 dermatopathologists, the WSI was shared with a third dermatopathologist. If the third dermatopathologist agreed with the first or second dermatopathologist, then the annotations were accepted using the data where agreement was found. If agreement was not found between any of the 3 dermatopathologists, the WSI was not included in the *Training Set*, as it did not meet the criteria for annotation.

The *Weakly Supervised Training Subset* consisted of 5161 WSIs from de-identified non-curated daily case specimens collected between April 15 and July 19, 2021 from PWL that met the aforementioned inclusion criteria. Each WSI in this set was independently reviewed by 2 concurring board-certified dermatopathologists and assigned a WSI-level diagnosis. The types of skin conditions covered in this set includes all skin lesion types in the *Supervised Training Set* along with more than 100 “other” skin conditions, including but not limited to various spindle cell lesions (e.g., dermatofibroma, neurofibroma, atypical fibroxanthoma, etc.), cysts (e.g., epidermal inclusion cyst, pilar cyst, etc.), vascular neoplasms, benign keratoses, sebaceous lesions, reactive and inflammatory conditions, and rare conditions that demonstrate morphologic features similar to those of the Mihm output diagnoses (Table S2).

Both training subsets included a diversity of sample types (i.e., approximately 90% shave or punch biopsies and 10% excisions). No WSIs in the *Training Set* were re-used within the *Validation Dataset* or the *Testing Dataset*.

#### Datasets: *Testing set*

The *Testing Set* consisted of 3821 WSIs from de-identified non-curated daily case (i.e., consecutive) specimens collected from July 20 to August 20, 2021 from PWL. The *Testing Set* was used for the ongoing testing of new iterations of Mihm, which was developed using the *Training Set*.

#### Validation dataset

Prior to releasing updated versions of Mihm into a production environment, each new version was proven to be equivalent or superior to the performance of the previous version on the *Testing Set* and *Validation Set* using a regression test. The *Validation Set* consisted of 250 curated de-identified WSIs, obtained from the tissue archives of PWL, that were selected by a team of expert dermatopathologists to represent a range of dermatopathological diagnoses reflecting the real-world clinical environment and was composed of: (i) 200 WSIs from consecutive specimens received at PWL that met the aforementioned inclusion criteria and (ii) 50 WSIs that represented difficult to diagnose WSIs or mimickers (Table S3), as they demonstrated morphologic features via H&E staining that were similar to features of Mihm output diagnoses. Mimickers were acquired from numerous clinics and referring providers.

### Mihm model training

**Fig. 3:**
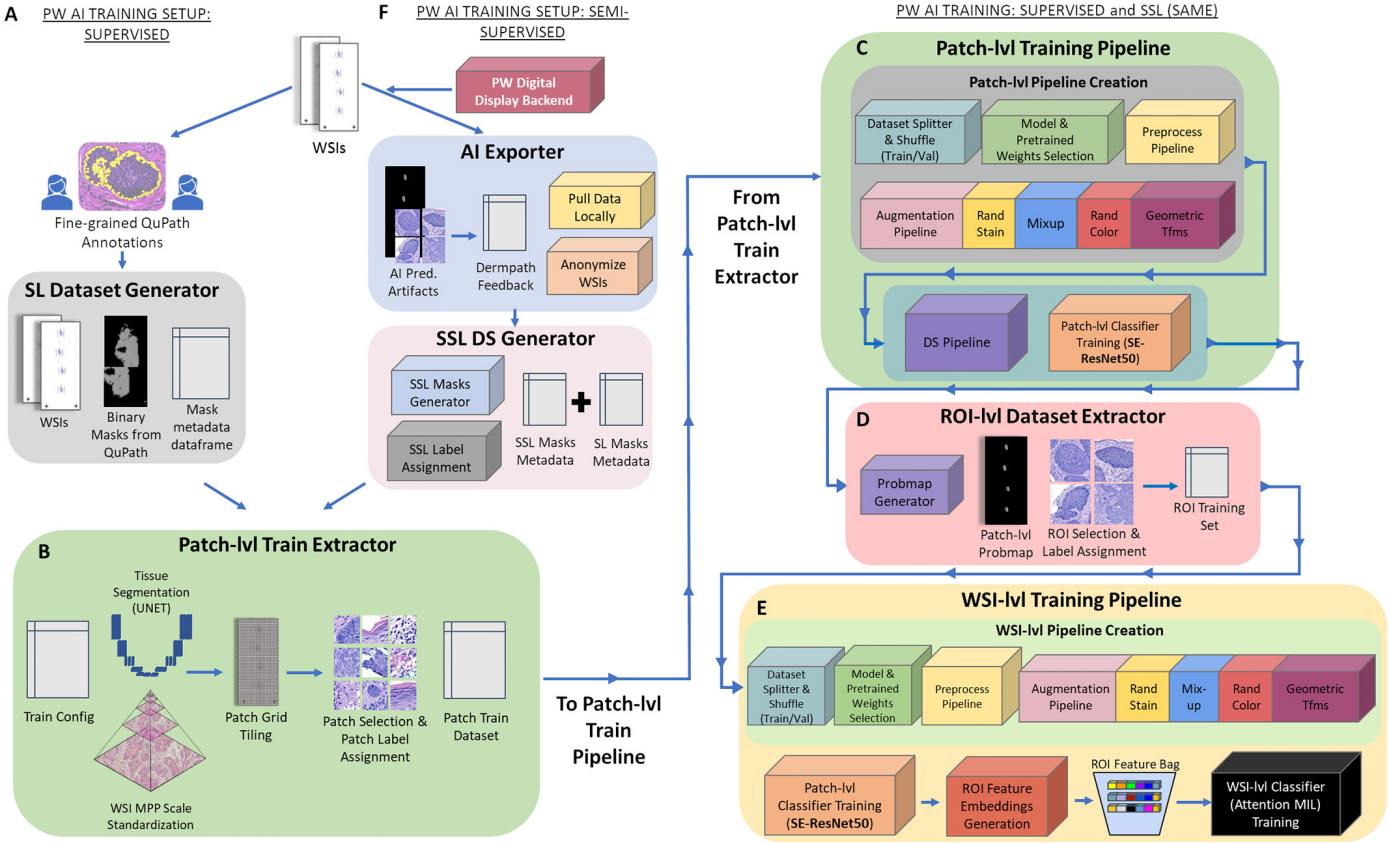
Mihm model training pipeline. Mihm’s skin lesion detection algorithm was trained to perform a comprehensive multi-level analysis of whole slide images (WSIs) to detect, localize, subtype, and provide margin status for multiple skin lesion types. Mihm model training occurs in 2 distinct phases: (i) supervised learning (SL) and (ii) semi-supervised learning (SSL). (A) Supervised training begins with the fine-grained annotation of WSIs in QuPath, resulting in binary masks with labels that corresponds to the annotated skin lesion and the coordinates of each mask that represent the region on the WSI. (B) Mask metadata is pulled from the SL Dataset Generator, a segmentation model (U-Net) is trained to segment tissue pixel regions of the WSI from background pixels. Tissue pixel regions are segmented via U-Net and each WSI is broken down into a grid of small window “patches” of equal size and is assigned a unique label so that Mihm learns to predict patches accurately for each lesion type. Lastly, the remaining tissue patches are assigned a label according to each patch overlap with the annotation mask region. (C) The patch-level dataset (i.e., patch image files and metadata) were used to train the patch-level model for each of 5 main skin lesion types. Separate binary image classifiers were trained for classifying patches into 1 of the 5 core skin lesion types as follows: melanocytic lesions, basal cell carcinoma, atypical squamous lesions, verruca vulgaris, and seborrheic keratosis. The datasets are first split into training (80%) and validation sets (20%), patch labels are binarized (i.e., 1 or 0), and trained via series of modules with operations chained together that are run in sequential order for 250 epochs on batches of the data as follows: random data augmentation, pixel normalization, model parameter update, and validation metrics calculation. These steps are repeated steps for each of the 5 specialized patch-level binary image classifiers. (D) Patch-level predictions are reconstructed as a probability map for the purposes of ROI selection in the ROI-level analysis to detect and highlight ROI-sized regions of the WSI that are suspicious of a particular skin lesion type. To prepare the dataset for ROI-level training, inference was run on all WSIs in the patch-level dataset by the probmap generator. A binary prediction was generated for all patches, and a probability map reconstructed for each WSI so that coordinates were assigned either a 1 or a 0 depending on the value of the prediction for patch window of the original WSI. After reconstructing the patch-level probability map for each WSI, larger ROI-size images are extracted from each WSI in the dataset with assigned binary labels to compose the ROI-level dataset for ROI-level training. (E) The ROI-level model employs SE-ResNet50 model architecture and begins with trained patch-level models. Here, the trained ROI-level model from the previous section is used to run inference on all the ROIs in the ROI-level dataset. The set of selected ROIs that we pre-extracted from each WSI in earlier steps were loaded as one batch, subject to pixel normalization, and then the ROI-level model run on the batch. Outputs (i.e., ROI feature embeddings) for each WSI utilizes attention-based deep multiple instance learning (MIL) network to train on “bags” of data, with just 1 bag per WSI. Separate WSI-level classifiers were trained for each of the 5 core lesion types and the WSI-level training loop was run by loading the ROI embedding bag and WSI-level label pairs for each WSI for each of the 5 specialized WSI-level binary image classifiers. (F) Semi-supervised learning is the second phase of Mihm’s model training. For the semi-supervised training set, automated annotation masks were generated in the same format as the masks that were hand-drawn in QuPath. Mihm’s full multi-level analysis, in which each of the model sets (i.e., Patch-level, ROI-level, and WSI-level) for each of the 5 main lesion types was run on each WSI in the weakly supervised training dataset from start to finish and the outputs used to auto-generate the SSL annotation masks and labels for each WSI, including patch-level probability maps, WSI-level predictions, and WSI-level labels (WSI ground truth provided by board-certified dermatopathologists). The SSL label assignment process, in which Mihm’s WSI-level prediction is compared to the WSI-level ground-truth diagnosis for each WSI, is repeated separately for each of the 5 main skin lesion types. SL and SSL mask metadata are merged into the semi-supervised training set and fed into the Patch-Level Train Extractor. WSI: whole-slide image, SL: supervised learning, SSL: semi-supervised learning, DS: dataset.

Mihm’s skin lesion detection algorithm was trained to perform a comprehensive multi-level analysis of all tissue areas of each H&E stained and scanned WSI, with the objective of detecting, localizing, subtyping, and providing margin status for multiple skin lesion types (Fig. 3). Mihm model training occurs in 2 distinct phases: (i) supervised learning and (ii) semi-supervised learning.

#### Patch-level training

In patch-level training, each WSI is broken down into a grid of small window “patches” of equal size (Table 2), in which each patch is assigned its own unique label according to the annotation mask so that Mihm learns to predict each patch region of the WSI accurately for each lesion type. As the size of the patch window is small relative to the full size of the WSI, patch-level predictions are reconstructed as a probability map to effectively localize detected skin lesions. However, while the patch-level model alone is not sufficient to accurately diagnose skin lesions at the WSI-level, patch-level predictions are used by Mihm for ROI selection in the next level of skin detection (See “ROI-level training”).

**Table 2:** Hyperparameters for patch-level training.

Hyperparameter	Value	
Epoch	250	
Batch size	64	
Validation data split	20%	

	**Mel, BCC, ASL**	**SK, VV**

Patch size	128 × 128 px	256 × 256 px
Base MPP	0.547619	2.1905

MPP: microns per pixel, Mel: melanocytic, BCC: basal cell carcinoma, ASL: atypical squamous lesion, SK: seborrheic keratosis, VV: verruca vulgaris, px: pixel.

##### Patch-level training: Data preprocessing

Data and annotations underwent a series of preprocessing steps prior to patch-level training as follows: (i) First, all supervised mask annotations from each WSI performed were exported from QuPath as binary mask files and were managed in a .csv file with the WSI name, mask file name, mask label name, mask coordinates, and other details. For each WSI, there was a set of binary masks with labels that corresponded to the annotated skin lesion and the coordinates of each mask that represented the region on the WSI (Fig. 3A). (ii) Next*,* a segmentation model (U-Net) was trained to segment tissue pixel regions of the WSI from background pixels (Fig. 3B). (iii) The original WSI file for each WSI at the patch-level was opened using OpenSlide (v3.4.1) and rescaled to a standardized resolution based on a hyperparameter “microns per pixel” (MPP) value for each WSI in the *Patch-Level Training Set* prior to segmentation by U-Net, and was subsequently broken down into a grid of small window patches of equal “patch size”, a hyperparameter set for each skin lesion type (Table 2; Fig. 3B). (iv) The tissue segmentation model was then run on the WSI to identify and remove all non-tissue region patches. (v) Lastly, the remaining tissue patches were assigned a label according to each patch overlap with the annotation mask region and all patches included in patch-level training for each WSI were saved as image files along with the metadata (e.g., patch coordinates, labels, etc.). The set of patch image files and patch metadata combined composed the *Patch-Level Training Dataset*.

##### Patch-level training: Training pipeline and modeling

Next, a model, loss function, optimizer, and learning rate were selected and a training pipeline prepared for specialized training of patch-level image classifiers for each skin lesion type that Mihm learned to detect. All modeling and training were performed using Google’s open source ML library Tensorflow v2.3.0.^66^

We used the CNN architecture squeeze and excitation network (SENet)^67^ SE-ResNet50, a standard ResNet50^68^^,^^69^ infused with “squeeze and excitation” blocks for the patch-level model. For each skin lesion type classifier, we began with a model that was pre-trained on ImageNet^70^, ^71^, ^72^ to leverage the learned parameters in lower layers of the network and subsequently converted the model to a binary classifier by replacing the last layer of the network with a one-unit dense layer (with randomly initiated parameters) and a sigmoid activation function. We then trained separate binary image classifiers specialized for classifying patches into 1 of the 5 core skin lesion types as follows: melanocytic lesion, BCC, ASL, SK, or VV. For the loss function, which measures the error rate of the model's predictions compared to the ground-truth label, we used the binary classification binary cross entropy.^73^ We used the Adam optimizer^74^ to optimize the learning rate of stochastic gradient descent and cosine annealing as the learning rate scheduler.^75^

Prior to using the *Patch-Level Dataset* to train the patch-level model for each of the 5 main skin lesion types, the dataset was first split by WSI into training (80% of WSIs) and validation sets (20% of WSIs) (Fig. 3C). Next, we used label binarization to transform the original assigned patch label annotations from string labels into a 1 or 0, thereby informing the model whether the patch was classed as positive or negative for each lesion type. After the *Patch-Level Dataset* and annotations were fully prepared for training, the training loop—a series of modules with operations chained together—was run in sequential order for 250 epochs (where an epoch is 1 full iteration of the dataset; Table 2) on batches of the data as follows: random data augmentation, pixel normalization, model parameter update, and validation metrics calculation (Fig. 3C).

For random data augmentation, we used a set of standard lossless data manipulation operations including Gaussian blur and noise, horizontal/vertical flips, rotations, cropping, affines (i.e., translation, scale, and shear), and color shifts (i.e., hue, saturation, and brightness) along with novel data augmentation techniques, including Mixup^76^ (i.e., linear interpolation of data and labels), and random stain mix (i.e., simulated random H&E stain mix). In *pixel normalization*, we normalized all pixel values in each RGB channel from integer values in the 0–255 range to float values in the range 0–1. The model parameter update is a standard ML model training loop and was used to send data batches through the model to generate outputs, calculate the model’s loss by comparing the model outputs to labels, calculate the gradients per the loss, and used an optimizer to optimize the model parameters using the gradients. At the end of each epoch of the *Training Set*, the validation metrics calculation ran an epoch of the *Validation Set* for the purposes of calculating validation metrics and saved the model parameters based on the optimal validation metrics. Notably, in the validation epoch, only pixel normalization was applied while both random data augmentation and model parameter update were bypassed.

The aforementioned steps were repeated for each of the 5 specialized patch-level binary image classifiers. Upon completion of training the patch-level models, Mihm proceeds with ROI-level training.

#### ROI-level training

As patch-level models alone are not sufficient to accurately diagnose skin lesions at the WSI-level, Mihm used patch-level models to generate localized probability maps for the purposes of ROI selection in this next level of our multi-level skin detection analysis. The objective of Mihm’s ROI-level analysis is to detect and highlight ROI-sized regions of the WSI that are suspicious of a particular skin lesion type, which are then used as input by Mihm’s highest-level analysis, performed by a WSI-level classifier, to finalize the WSI-level diagnosis.

##### ROI-level training: Probmap generation

To prepare the dataset for ROI-level training, inference was run on all WSIs in the *Patch-Level Dataset* in a process called probmap generation (Fig.3D). In the probmap generation workflow, predictions were first generated for all tissue patches in each WSI by the Patch-Level Inference Pipeline as follows: WSI was loaded in OpenSlide, rescaled to a standardized resolution using a predefined MPP hyperparameter (Table 2), background patches were filtered out using the trained tissue segmentation model (U-Net), patch pixels were normalized, and the trained patch-level model was run on all remaining tissue patches to generate a binary prediction for each patch. For each patch, we applied a fixed set of augmentation methods to the original image (including vertical and horizontal flips and rotations) to generate multiple variants of the image, and then took the mean prediction across the batch of images to assign a final prediction for the patch (a technique known as “test time augmentation”). Once a binary prediction was generated for all patches, a probability map was reconstructed for each WSI so that coordinates were assigned either a 1 or a 0 depending on the value of the prediction for patch window of the original WSI. For a patch to be considered a positive prediction, we used a fixed hyperparameter threshold of 0.8 (i.e., the prediction float value must be >0.8 to be considered a positive patch).

##### ROI-level training: ROI selection and dataset generation

After reconstructing the patch-level probability map for each WSI, larger ROI-size images were extracted from each WSI in the dataset with assigned binary labels for both the positive class (ROI contains skin lesion type being detected) and negative class (ROI does not contain the skin lesion being detected), and were subsequently used as the *ROI-Level Dataset* for ROI-level training (epoch: 250; batch size: 2; ROI size: 1024 × 1024 px [20x magnification equivalent] for Mel, BCC, and ASL and 512 × 512 px [5x magnification equivalent] for SK and VV). First, post-processing was run on the patch-level probability map to remove highly probable false-positive predictions. Next, all remaining regions of the probability map were collected and sorted by area in which the top N regions, where N is a hyperparameter and top N regions refer to regions with the highest density of positive predictions, were selected as ROIs for training and the ROI window placed at the center of each region. The label assigned for each ROI corresponded to the WSI-level label such that if the WSI contained any patches with annotations of the positive class, the WSI and ROIs were assigned a positive label. If not, the WSI and ROIs were assigned a negative label. If no ROIs were detected on a particular WSI in this step, a random ROI selection was performed. Once ROIs were extracted and assigned labels for all WSIs in the dataset, the *ROI-Level Training Set* can be used for training in the ROI-Level Training Pipeline.

##### ROI-level training: Training pipeline and modeling

Aside from the difference in data inputs and labels, ROI-Level Training Pipeline and modeling was similar to the Patch-Level Training Pipeline (See “Patch-level training: Training pipeline & modeling”). ROI-level training also required a model, loss function, optimizer, and learning rate schedule, as well as a training pipeline for specialized training of an image classifier for each lesion type that Mihm detected on ROI-sized regions of the WSI (Fig. 3).

As with patch-level models, our ROI-level model employed the same network type (CNN) and model architecture (SE-ResNet50). However, instead of starting from the model pre-trained on ImageNet, the ROI-level model began with trained patch-level models, and thus no further modifications to the model were needed. Like patch-level training, separate binary image classifiers were trained for each of the 5 core skin lesion types (i.e., melanocytic lesions, BCC, ASL, SK, and VV). Loss function, optimizer, and learning rate schedule were identical to those of patch-level training.

As patch-level models were used in ROI-level training and learned parameters were trained on the *Patch-Level Dataset*, there was no random split of the dataset by WSI. Instead, the *Training* and *Validation Sets* defined in the patch-level training process were preserved. Likewise, the ROI-training loop was identical to that of patch-level training (Fig. 3E). The steps outlined in this section were repeated for each of the 5 specialized ROI-level binary image classifiers prior to WSI-level training.

### WSI-level training

After training the 5 specialized patch-level models, which provided localized clusters of detected skin lesions, and the 5 specialized ROI-level models, which highlighted key diagnostic ROI-sized regions of the WSI, Mihm could finalize the top-level, WSI-level based diagnosis.

#### WSI-level training: Dataset generation

To prepare the dataset for WSI-level training, the trained ROI-level model from the previous section was used to run inference on all ROIs in the *ROI-Level Dataset* in the ROI-Level Inference Pipeline (Fig. 3E).

In the ROI-Level Inference Pipeline, we iterated through each WSI in the *ROI-Level Dataset*. However, instead of loading WSIs, the set of selected ROIs that we pre-extracted from each WSI in earlier steps was loaded as one batch, subject to pixel normalization, and then the ROI-level model run on the batch. Then, instead of employing the standard process of generating predictions from the final layer of the trained ROI-level model, we instead saved the outputs of the penultimate layer, specifically the global average pooling (GAP) layer,^77^ which we refer to as ROI feature embeddings. Upon completion of the ROI-level Inference Pipeline, we had 1 ROI feature embedding (with batch size equal to the number of ROIs) for each WSI in the dataset, while the *WSI-Level Dataset* consisted of the ROI embeddings and WSI-level label pairs. Together, these features composed the *WSI-Level Training Set* for training in the WSI-Level Training Pipeline.

#### WSI-level training: Training pipeline

The WSI-level training process differs from patch- and ROI-level trainings because we trained on feature embeddings as opposed to images, with one data point per WSI as opposed to thousands of patches or dozens of ROIs. Only at this stage of the analysis is there enough information to provide the final WSI-level predicted diagnosis for each WSI.

The WSI-level model utilized attention-based deep MIL network,^78^ which is a form of weakly supervised learning that trained on “bags” of data, where a bag was a batch of ROI feature embeddings with just 1 label assigned to the bag (1 bag per WSI) instead of multiple unique labels for each instance in the bag. As with ROI-level training, separate WSI-level classifiers were trained for each of the 5 core lesion types: melanocytic lesions, BCC, ASL, SK, and VV. The loss function, optimizer, and learning rate schedule used in WSI-level training were identical to those used both patch- and ROI-level trainings. Additionally, as with ROI-level training, there was no random split of the dataset for WSI-level training.

The WSI-level training loop is vastly simplified, as iterations through the *WSI-Level Dataset* were performed by loading the ROI embedding bag and WSI-level label pairs for each WSI and then applying the model parameter update and validation metrics calculation as previously performed in the other training pipelines. Finally, these steps were repeated for each of the 5 specialized WSI-level binary image classifiers.

### Semi-supervised training

The second phase of Mihm model training, SSL (Fig. 3F), transitions from training a set of models using SL methods to fine-tuning and improves upon these models by leveraging the much larger *Weakly Supervised Training Subset*, consisting of 5161 WSIs. Unlike the *Supervised Training Subset* (i.e., curated specimens with fine-grained, hand-drawn localized masks), the *Weakly Supervised Training Subset* was composed of non-curated daily case specimens that were assigned a WSI-level diagnosis and covered a much wider range of “other” skin conditions (>100) beyond the 5 core lesion types that Mihm was trained to detect in the first phase of model training (Table S2).

#### Semi-supervised training: Dataset preparation

**Fig. 4:**
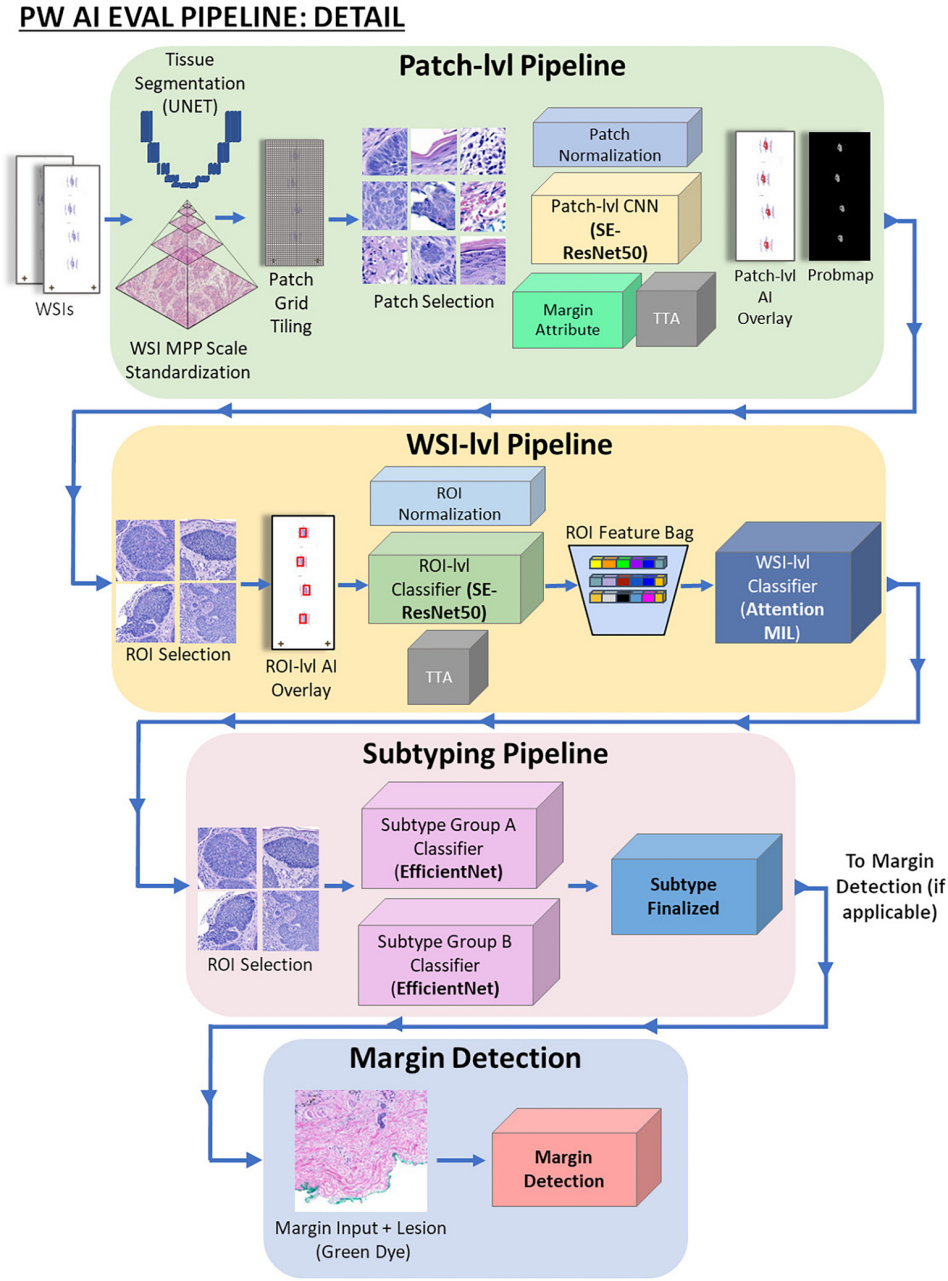
Mihm Evaluation Pipeline. In the Patch-Level Pipeline, tissue pixel regions are segmented via U-Net. Each WSI is broken down into a grid of small window “patches” of equal size and is assigned a unique label based on the model prediction. Here, in the Patch-Level Pipeline, which is modified to include margin patch assignments, all margin patches of all tissue sections of the WSI are identified and localized, and subsequently used to perform a margin status analysis. Once patch-level predictions are generated, the probability map for ROI selection is reconstructed. The Subtyping Dataset relies on WSIs from the semi-supervised dataset with positive labels for the lesion types that require subtyping (i.e., melanocytic lesions, atypical squamous lesions [ASL], and basal cell carcinoma [BCC]). For the data inputs, we ran the subtyping analysis on the same ROIs from the ROI-level dataset and then expanded the ROI search to the edges of the detected lesion regions. To properly assign ROI subtype labels to cases with multiple subtypes in the weakly supervised dataset, a team of board-certified dermatopathologists reviewed pre-extracted ROI images and assigned the unique subtype to each. The CNN model architecture ConvNeXt Tiny variant pre-trained on ImageNet-21K was used for the subtype model. The subtype training loop (i.e., random data augmentation, pixel normalization, model parameter update, and validation metrics calculation) was repeated for each of the lesion subtype binary image classifiers. The subtyping inference module is dependent on the results of the WSI-level core lesion analysis and is only run if Mihm detects a positive result for melanocytic lesions, ASL, or BCC. If the WSI is detected as positive for one of these lesion types, the associated subtyping models and corresponding subtyping analysis are run accordingly. For each positive lesion type, ROI-level images that are extracted in the ROI selection process from the patch-level probability maps are fed into the Subtyping Inference Pipeline, which searches for the most severe subtype in the WSI by systematically running each of the subtyping classifiers in order of maximum severity. If after all subtyping models are run and no subtype is detected at any severity level, the WSI-level subtype result is deemed inconclusive. For margin detection, a margin map is reconstructed according to each patch’s margin attribute recorded in margin patch assignment. With the patch-level prediction probability map and margin map combined as inputs, the margin status analysis is performed to determine overlap between the detected lesion and margin tissue. Based on the results of this analysis, we can assign the proposed margin status to the WSI. Margin status is finalized after the WSI-level lesion type and subtype are finalized according to the criteria for assigning margin status. WSI: whole slide image, ROI: region of interest, MIL: multiple instance learning, TTA: test time augmentation.

For the *Semi-Supervised Training Set*, we generated automated annotation masks in the same format as the masks that were hand-drawn in QuPath. First, Mihm’s full multi-level analysis was run in the Evaluation Pipeline (Fig. 4) on each WSI in the *Weakly Supervised Training Subset* from start to finish for each of the model datasets (i.e., *Patch-Level*, *ROI-Level*, and *WSI-Level*) for each of the 5 main lesion types. The outputs were then used to auto-generate the SSL annotation masks and labels for each WSI, including patch-level probability maps, WSI-level predictions, and WSI-level labels (WSI ground truth provided by a board-certified dermatopathologist). The SSL label assignment process, in which Mihm’s WSI-level prediction is compared to the WSI-level ground truth diagnosis for each WSI, was repeated separately for each of the 5 main skin lesion types as follows: (i) In the case of a *true-positive (TP)*, where the WSI has a WSI-level label that is positive for 1 of the 5 main skin lesion types and Mihm’s WSI-level prediction is also positive for the matching skin lesion type (e.g., Mihm predicted positive for BCC with WSI-level label of BCC), the SSL label was assigned a positive label for the lesion type. (ii) In the case of a *true-negative (TN)*, where both the WSI-level label and Mihm’s WSI-level prediction were negative for the same skin lesion type (e.g., Mihm predicted negative for BCC and the WSI-level label was negative for BCC/positive for any condition other than BCC), the SSL label was assigned a negative label for the lesion type (i.e., “Not XX lesion”). (iii) In the case of a *false-positive (FP)* where Mihm’s WSI-level prediction was positive for a skin lesion type, but the WSI-level label was negative (i.e., Mihm predicted positive for BCC and the WSI-level label was negative for BCC and positive for any condition other than BCC), the SSL label was also assigned a negative label for the lesion type (i.e., “Not XX Lesion”). (iv) In the case of a *false-negative (FN)*, where the WSI-level label is positive for the skin lesion type and Mihm is negative for the same lesion type (i.e., Mihm predicted negative for BCC and the WSI-level label was positive for BCC), the WSI is excluded from the SSL auto-generated mask process and flagged for creation and review of ROIs. The WSIs that are part of the SSL label assignment process (steps i–iv) described above were then reviewed by a second dermatopathologist. If there was agreement between the dermatopathologists, the WSIs and accompanying masks were included in the *Weakly Supervised Training Set*. If the 2 dermatopathologists disagreed, then the WSI was excluded.

SSL label assignment yields a dataset of WSIs in which each WSI has its own set of auto-generated SSL masks for each of the 5 main skin lesion types. These SSL masks are identical to the patch-level probability maps with the exception that they have been assigned ground-truth labels. As our training pipelines do not support the direct use of patch-level probability maps as annotations for training, the masks must be converted to the same format used by QuPath in the supervised dataset as follows: (i) the corresponding SSL mask and assigned SSL label were loaded for each WSI and skin lesion type, (ii) post-processing on the SSL mask was run to remove any low-confidence/noisy regions, (iii) the cleaned SSL mask was rescaled to the standard scale used by the QuPath generated masks, (iv) each region of the SSL mask was saved as a separate SSL annotation file and the same metadata information used in the *Supervised Dataset* was generated for each SSL annotation as an entry in a .csv file used for managing the SSL annotations. Following SSL annotation, the SSL dataset was fully prepared for training in the same format as the *Supervised Dataset* (i.e., has a set of binary masks for each WSI with labels that correspond to the annotated skin lesion and the coordinates of each mask representing the region on the WSI).

#### Semi-supervised training: Training pipeline

The first step in the SSL Training Pipeline was to merge the *Supervised Training Subset* with the *Weakly Supervised Training Subset* to form a consolidated dataset: the *Semi-Supervised Training Set*. When merging, it was important to retain the original dataset split from the *Supervised Training Subset* that was previously used to train Mihm while performing a new split of the *Weakly Supervised Training Subset* based on the order in which it was originally processed by the lab (i.e., according to case_id). Splitting is not random so that the *Validation and Test Sets* are as representative as possible of the true test environment, in which routine cases are processed by the lab.

Semi-supervised training takes place once *Supervised* and *Weakly Supervised Datasets* are combined into 1 *Semi-Supervised D*ataset. As our training pipelines are standardized across all levels (i.e., patch-level, ROI-level, and WSI-level), the workflow for training each of the models for each of the 5 lesion types in the second phase of training is the same as in the first phase (i.e., supervised training). Thus, for each skin lesion type and for each level of analysis, the model was trained on the *Semi-Supervised Dataset* starting from the previously trained parameters.

### Subtyping: Overview

**Fig. 5:**
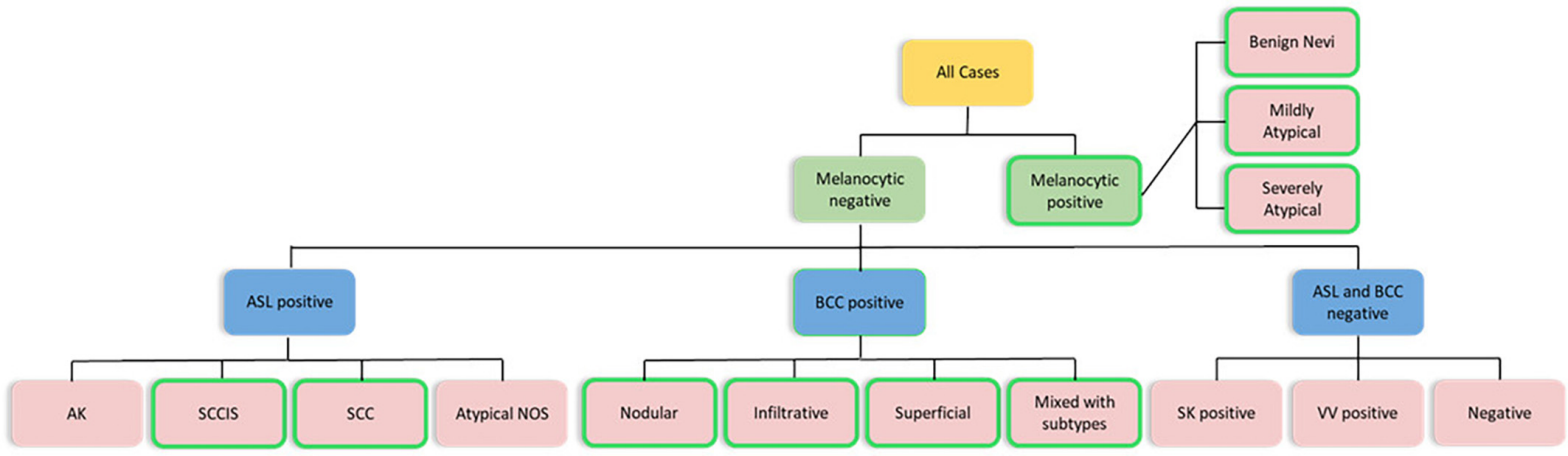
Mihm subtyping algorithm design. Lesion types detected by Mihm are as follows: Melanocytic lesions with subsequent subtyping into benign nevi, mildly atypical, or severely atypical; basal cell carcinoma (BCC) with subsequent subtyping into superficial, nodular, infiltrative, or mixed subtypes; atypical squamous lesions with subsequent subtyping into invasive squamous cell carcinoma (SCC), squamous cell carcinoma in situ (SCCIS), actinic keratosis (AK), and atypical squamous lesion (ASL) not otherwise specified (NOS); seborrheic keratosis (SK); and verruca vulgaris (VV). Green outlines around lesion types indicate lesion types for which margin detection was performed. Margin detection was applied to the following lesion types: melanocytic (all subtypes), SCCIS (ASL +), SCC (ASL +), nodular (BCC +), infiltrative (BCC +), superficial (BCC +), and mixed with subtypes (BCC +), as indicated by green outlining.

Mihm not only performs a comprehensive multi-level analysis of each case specimen(s) that detects and localizes malignant and/or atypical lesions, but also provides a qualitative assessment of the lesion’s specific subtype as follows: (i) melanocytic lesions with margin status and subsequent subtyping into benign nevi (i.e., junctional nevus, dermal nevus, compound nevus, halo nevus, neurotized nevus, lentiginous nevus, blue nevus, and recurrent nevus), mildly atypical melanocytic lesions (i.e., junctional dysplastic nevus mild, junctional spitz nevus, dermal spitz nevus, dysplastic nevus dermal banal, dysplastic nevus dermal low-grade, and atypical spitz nevus/tumor), and severely atypical melanocytic lesions (e.g., junctional dysplastic nevus moderate, junctional dysplastic nevus severe, severely atypical junctional melanocytic proliferation, invasive melanoma, melanoma in-situ, and melanoma in-situ lentigo maligna); (ii) BCC with margin status and subsequent subtyping into superficial, nodular, infiltrative, or mixed subtypes; (iii) ASL and subsequent subtyping into invasive SCC, SCCIS, AK, with margin status for SCC and SCCIS; (iv) SK; and (v) VV. The algorithm design for subtyping in the Evaluation Pipeline can be seen in Fig. 5.

#### Subtyping: Dataset preparation

The *Subtyping Dataset* relied on WSIs from the *Semi-Supervised Dataset* with positive labels for the lesion types that required subtyping (i.e., melanocytic lesions, ASL, and BCC). From this new subset, we prepared an *ROI-Level Dataset* for training and analysis that was unique from the previously described ROI-level process. For data inputs, we ran the subtyping analysis on the same ROIs from the *ROI-Level Dataset* and then expanded the ROI search to the edges of the detected lesion regions. The motivation for this expansion is the need for sufficient context of lesion growth patterns throughout the tissue sections to detect the level of severity/invasiveness of subtypes.

For ROI label assignment, we could not merely use the binary labels previously assigned for the core lesion types, as Mihm’s subtype analysis needed to detect lesion subtype from a set of multiple severity levels at a more granular level. This was straightforward for the *Supervised Dataset*, as the supervised annotation masks performed in QuPath were already defined at the subtype level, and thus assigned the subtype label to each ROI in the *Supervised Dataset* accordingly. However, for the *Weakly Supervised Subset*, with only WSI-level labels as ground truth, the lesion subtype label could not map to specific ROIs. To properly assign ROI subtype labels to cases with multiple subtypes in the *Weakly Supervised Subset*, a team of board-certified dermatopathologists reviewed pre-extracted ROI images and assigned the unique subtype to each. Once ROIs were extracted and assigned labels for all WSIs in the dataset, the *Subtyping Training Set* was completed and ready for the Subtyping Training Pipeline.

#### Subtyping: Training pipeline and modeling

For the subtype model, we used the CNN model architecture ConvNeXt, specifically the ConvNeXt Tiny variant pre-trained on ImageNet-21K.^79^ As with the patch-level model, we modified the final layer of the network to a binary output with sigmoid activation starting from randomly initialized parameters. As the *Subtyping Dataset* only included WSIs with a positive label, the task of the subtyping model was focused on differentiating the subtype for the specific lesion subtype being trained. The same model configuration was used to train separate binary image classifiers for each of the lesion subtypes. The loss function, optimizer, and learning rate schedule were identical to those of previously described training pipelines. We also preserved the same split of *Training* and *Validation Sets* used previously for the semi-supervised training of the core lesion types. Additionally, the subtype training loop relied on the modules used in the other training pipelines (i.e., random data augmentation, pixel normalization, model parameter update, and validation metrics calculation). These steps were repeated for each of the lesion subtype binary image classifiers.

#### Subtyping: Inference pipeline

The Subtyping Inference Pipeline transforms ROI-level images extracted from patch-level probability maps into ROI-level subtype detections and ultimately a WSI-level subtype analysis. The subtyping inference module is dependent on the results of the WSI-level core lesion analysis and was only run if Mihm detects a positive result for melanocytic lesions, ASL, or BCC, whereupon the associated subtyping models and corresponding subtyping analysis were run accordingly. For each positive lesion type, ROI-level images that were extracted in the ROI selection process from the patch-level probability maps were fed into the Subtyping Inference Pipeline, which searches for the most severe subtype in the WSI by systematically running each of the subtyping classifiers in order of maximum severity (i.e., for ASL, the analysis would first perform a search for invasive SCC). If the most severe subtype was detected, the WSI-level subtype was assigned to the max severity level accordingly. Otherwise, the subtype inference analysis continued to the next level of severity until a match was found. If after all subtyping models were run and no subtype was detected at any severity level, the WSI-level subtype result was deemed inconclusive.

#### Margin detection: Overview

Mihm has also been designed to perform margin detection to inform if a given lesion has a positive or negative margin (i.e., extends to the non-epidermal edges). Specifically, margin status was detected for all melanocytic lesions, all BCC, and ASL subtypes SCCIS and SCC invasive. Mihm does not provide margin status for ASL subtype AK or for benign lesion types SK and VV.

#### Margin detection: Margin patch assignment

First, areas of tissue sections in the WSI that have been marked with green dye in the inking process were localized. Owing to the relatively small size of the inked tissue edges, we found that it was most effective to identify and assign margins at the patch-level using our existing Patch-level Inference Pipeline with a few minor modifications. We incorporated the margin patch assignment into our Patch-Level Inference Pipeline as follows: as each batch of patches was loaded for analysis by the patch-level model, we converted the images from RGB to hue, saturation, value (HSV) colorspace to determine if each patch was a margin patch based on the range of pixel intensity values in HSV. The result of the patch’s margin check (i.e., true or false) was recorded as an attribute for further analysis.

#### Margin detection: Margin status analysis

After the modified Patch-Level Inference Pipeline, which was modified to include margin patch assignments, all margin patches of all tissue sections of the WSI were identified and localized, and subsequently used to perform a margin status analysis. As described in earlier sections, once patch-level predictions are generated, the probability map for ROI selection is reconstructed. In margin detection, we included an additional step to also reconstruct a margin map according to each patch’s margin attribute recorded in margin patch assignment (See “Margin detection: Margin patch assignment”). With the patch-level prediction probability map and margin map combined as inputs, the margin status analysis was performed to determine overlap between the detected lesion and margin tissue. Based on the results of this analysis, we were able to assign the proposed margin status to the WSI. The margin status was finalized in a later step once the WSI-level lesion type and subtype were finalized according to the criteria for assigning margin status as either positive or negative.

### Mihm: Evaluation pipeline

Once Mihm was fully developed, it was used in practice to finalize Mihm’s overall WSI-level prediction using a chain of hierarchical modules, each with its own specialized multi-level analysis, all run in sequential order with a set of logical rules, called the Evaluation Pipeline. As shown in Fig. 5, the first module is run for melanocytic lesions and runs the entire multi-level analysis as follows: patch-Level, ROI-Level, WSI-Level, subtyping, and margin status. If the module for melanocytic lesions resulted in a WSI-level positive prediction, the Evaluation Pipeline discontinued further analyses and returned the results for melanocytic lesion, associated subtype, and margin status. Otherwise, the Evaluation Pipeline continued to the next module for BCC and ASL. If Mihm’s WSI-level prediction was not positive for either BCC or ASL and the case specimen is not from an excision, the module was run for benign lesions SK and VV.

### Continual learning: PW digital display, dermatopathologist feedback, and AI exporter

The trained Mihm algorithm was used to run the full Evaluation Pipeline on WSIs, yielding AI classification results. Those AI results were coupled with dermatopathologist feedback within the PW digital display Backend (Fig.3F) and exported using the AI Exporter module to create a continuous feedback loop for ongoing AI updates and releases. To do this, the AI Exporter automatically pulls WSIs from the PW digital display Backend as .csv files, which include the AI predictions, slide identification (ID), slide classification ID, diagnostic result, subtype, WSI-level prediction, remarks, primary dermatopathologist label, secondary dermatopathologist label, secondary dermatopathologist reviewer, diagnosis mismatch and clinically significant diagnostic mismatches, the data source for each slide (i.e., dermatopathologist or AI feed), and other associated data. This iterative feedback loop (Fig. 1) enables fast and efficient scaling of the ML dataset while specifically targeting areas of weakness in Mihm to make it more robust for an ever-growing range of skin conditions from routine daily caseload.

### Integrations (UX/UI)

#### Front-end development

The front-end is built with React, a declarative JavaScript library used to build high-performance and responsive user interfaces (UI).^80^ We built on this with numerous functional programming principles, as opposed to object-oriented programming principles.

#### Retrospective review

The retrospective review UI provides a simple interface to allow a group of pathologists to evaluate the AI diagnosis of a case after the case has been already finalized by a pathologist to create a process for an additional review, where an additional pathologist can review the original pathologist’s diagnosis and the AI diagnosis to determine: (i) if there was a mismatch in diagnosis results, (ii) which process (human pathologist or AI) provided the more accurate diagnosis, (iii) if the diagnosis mismatch was clinically significant, (iv) if additional peer review is required, and (v) if there was an image or slide quality issue (e.g., blurry image, tiled image, fragmented, folded tissue, etc.).

#### AI results and overlay

**Fig. 6:**
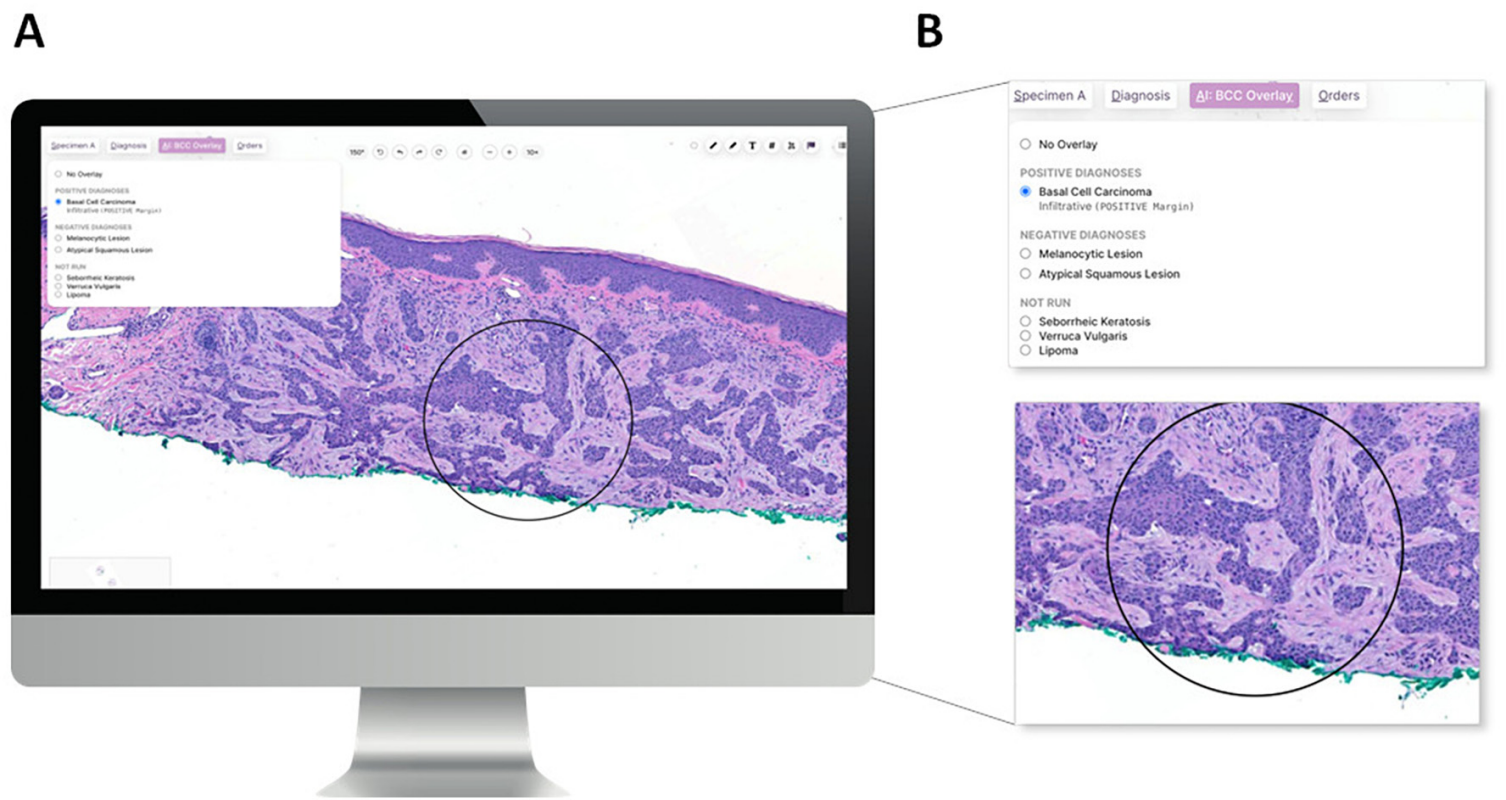
Example of AI overlay in the front-end digital display following the analysis of a whole slide image by the computational pathology algorithm Mihm Diagnostic Assist. AI overlay is selected by the pathologist in the digital display. (A) Pathologist view of the front-end digital display. The menu (inset) shows the digital display with “BCC Overlay” enabled and indicates the lesion type (i.e., BCC), subtype (i.e., infiltrative), and margin status (i.e., positive). (B) Close-up view of (i) the AI Overlay Dialog Box and (ii) a region of interest identified by the AI, which overlaps the green (i.e., non-epidermal) margin, thereby indicating a positive margin.

The overlay is displayed to the pathologist as a circle that identifies ROIs that the AI used to predict the diagnosis (Fig. 6). This simple overlay is purposefully pared back according to dermatopathologist input from any patch- or heatmap-based visualizations to reveal as much of the slide as possible without obscuring any relevant portions. Notably, the result of the AI is not revealed until the pathologist explicitly interacts with the digital display to open “AI: Overlay” in the menu, which reveals the full menu of the various lesion line algorithms of Mihm.

### Statistical analyses

The success of the AI model was evaluated by calculating the sensitivity (TP)/(TP+FN), specificity (TN)/(TN+FP), as well as the area under the curve (AUC), where the AUC is the area under the ROC (receiver operating characteristic) curve. The ROC curve is plotted by the true-positive rate (TPR) false-positive rate (FPR) at a range of thresholds between 0 and 1, and were calculated for each diagnosis category (BCC, ASL, melanocytic, VV, and SK). Continuous variables are presented as means and 95% confidence intervals (CIs).

## Results and discussion

In the present study, we developed a novel and robust end-to-end dermatopathology system that includes: (i) access to diverse, high-quality samples; (ii) standardized tissue processing; (iii) a comprehensive, multi-level AI model that seamlessly combines both SL and SSL to detect, localize, subtype, and provide margin status for 5 classes of skin lesions; and (iv) a front-end digital display for dermatopathologists to interact with samples and provide feedback on AI predictions that cycle back into the SSL Pipeline for ongoing AI development. During AI development, preliminary experiments determined that reliable localization was not possible without a supervised component (data not shown). In contrast, a model that relied solely on SL would not be well generalized to large numbers of cases and would thus also underperform on localization. As a result, Mihm evolved to combine both SL pre-training and SSL, yielding a high-fidelity and generalizable model that can also subtype and effectively localize ROIs within tissue sections, allowing for the reliable determination of margin status where applicable.

**Fig. 7:**
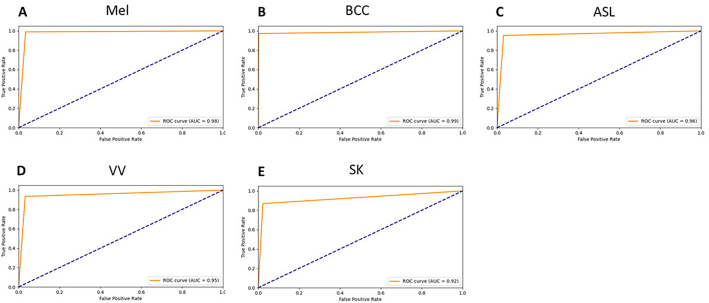
Receiver Operating Characteristic (ROC) curves for the 5 core lesion types detected by Mihm in the present study. (A) Melanocytic lesions (Mel). (B) Basal cell carcinoma (BCC). (C) Atypical squamous lesions (ASL). (D) Verruca vulgaris (VV). (E) Seborrheic keratosis (SK).

As of March 5, 2022, the *Supervised Training Subset* included a total of 106 692 individual, hand-drawn annotations for supervised pre-training. The innovative and seamless integration of the large-scale *Supervised Training Subset* (2188 WSIs) and the diverse *Weakly Supervised Training Subset* (5161 WSIs) into the *Semi-Supervised Training Set* enabled Mihm to: (i) effectively screen for melanocytic lesions and (ii) deliver a definitive diagnosis for commonly encountered non-melanocytic lesions with a high degree of accuracy. To avert the potentially negative effects of selection and verification bias during training, the *Training Set* included mimickers, special cases, as well as biopsies and excisions. Sensitivity, specificity, and the AUC were used to evaluate the overall performance of Mihm. The performance of Mihm on the *Testing Set* (3821 WSIs) can be seen in Table 3. Notably, Mihm achieved a sensitivity of 98.91% (95% CI: 98.27%, 99.55%) for melanocytic lesions, as well as sensitivities of 95.26% (95% CI: 93.79%, 96.73%) and 97.24% (95% CI: 96.15%, 98.33%) for ASL and BCC, respectively. The area under the ROC curves—which demonstrates the relationship between sensitivity and specificity—highlights the performance of the AI model for each of the 5 classifiers in detecting their respective skin lesion types (Fig.7). Additionally, the AUC values for melanocytic lesions, ASL, and BCC were 0.98 (95% CI: 0.973, 0.982), 0.96 (95% CI: 0.954, 0.967), and 0.99 (95% CI: 0.981, 0.989), respectively.

**Table 3:** Performance characteristics and 95% confidence intervals of Mihm using the Testing Set.

Lesion type (3821 WSIs)	Sensitivity	Specificity	AUC	Sensitivity 95% CI	Specificity 95% CI	AUC 95% CI
Mel	98.91%	96.61%	0.98	[98.27%, 99.55%]	[96.02%, 97.20%]	[0.973, 0.982]
BCC	97.24%	99.78%	0.99	[96.15%, 98.33%]	[99.60%, 99.95%]	[0.981, 0.989]
ASL	95.26%	96.82%	0.96	[93.79%, 96.73%]	[96.13%, 97.51%]	[0.954, 0.967]
VV	93.50%	96.79%	0.95	[89.14%, 97.86%]	[95.93%, 97.65%]	[0.941, 0.962]
SK	86.91%	97.74%	0.92	[82.13%, 91.69%]	[96.99%, 98.49%]	[0.910, 0.936]

WSI: Whole slide image, BSS: basal cell carcinoma, ASL: atypical squamous lesion, AK: actinic keratosis, SCC: squamous cell carcinoma, SCCIS: squamous cell carcinoma in-situ, Mel: melanocytics, AUC: area under the curve.

Not only does Mihm’s full multi-level analysis, trained on a large-scale and diverse dataset, classify the 5 main types of skin lesions at the WSI level with a high degree of sensitivity, but the creation of a novel *Subtyping Training Set* enabled us to train a specific pipeline for subtyping applicable lesion types. Furthermore, Mihm also assigns patch-level predictions at its most granular level, which can be converted to a probability map for the localization of lesions and margins on the WSI at the ROI-level. During the development of margin detection, we investigated numerous methods of detecting margin status, such as first training an epidermis detection algorithm to identify the epidermis in each tissue section and then taking the opposite tissue edge as the margin (data not shown). However, our experiments showed that these methods were often unreliable owing to irregularities in the appearance of the epidermis or the absence of the epidermis in a tissue section. As we also process FF and FFPE tissues in our facility, we determined that Mihm can be trained to detect green dye applied to the non-epidermal surface during tissue grossing to determine the distance of the lesion to the margin and detect any overlap between positive tumor patches and the tissue margin, thereby automating margin status (i.e., positive or negative margin) at non-epidermal edges. As positive margins are known to be associated with significantly lower survival in melanomas,^81^ and accurate determination of margin status is critical to determining the associated patient therapy of numerous types of skin lesions, this feature has the potential to substantially change the course of patient care. These qualitative features, combined with the front-end digital display and AI overlay to localize diagnostic ROIs, may serve as a diagnostic assist to further streamline pathology workflows and increase efficiency and confidence for dermatopathologists.

Currently, efficiency is hampered in traditional clinical workflows by factors such as increased caseloads, a global shortage of dermatopathologists, and high discordance rates.^22^^,^^23^^,^^47^^,^^82^ Moreover, even expert dermatopathologists express apprehension at diagnosing melanocytics using non-digital methods, which could ultimately impact diagnostic confidence and delay diagnosies.^23^^,^^83^^,^^84^ In contrast, studies that have evaluated the potential of AI in clinical pathology workflows have shown that the use of AI significantly decreases time to diagnoses and increases pathologist accuracy and diagnostic confidence.^85^^,^^86^ Likewise, our preliminary results show significant reductions in the average time spent by pathologists per specimen and concomitant increases in diagnostic confidence (*p* < .001) when AI and the PW front-end digital display were incorporated into pathology workflows (*manuscript in prep*).

The vital role of dermatopathologists in developing AI-based systems for clinical workflows has been widely recognized. A systematic review that examined the role of dermatologists in developing AI models for screening and assessing skin cancer using dermoscopic or gross specimen lesions underscores the importance of expert, specialized physician input and oversight for improved outcomes.^87^ This review determined that dermatologists are underrepresented in publications describing these technologies that are so applicable to their field and that dermatologist involvement is crucial to the design of clinically relevant and effective models.^87^ Likewise, involving expert physicians in AI development also alleviates another considerable challenge that exists in digital dermatopathology: the availability of high quality and diverse datasets.^20^^,^^44^^,^^47^^,^^62^ In the present study, expert knowledge drove features such as UX/UI design and AI overlay and was instrumental in helping to filter outputs, thereby overcoming limitations in interpretability, leading to increased confidence in AI results. The involvement of dermatopathologists at every step of model development provided constant and valuable feedback for both back- and front-end processes. Such close collaboration is vital for developing and deploying technologies into clinical practice.

Despite the robustness of our end-to-end system, there are some potential limitations. For example, Mihm does not currently detect Merkel cell carcinoma, a rare and aggressive cutaneous neuroendocrine tumor that can simulate and be mistaken for BCC. In addition, Mihm does not yet detect many other benign and malignant lesions or perform grading of SCC to determine differentiation. Future versions of Mihm will address these shortcomings by expanding the detection algorithms to include a Merkel cell screener for BCC cases, as well as to detect other lesion types. While Mihm has one of the largest training, validation, and testing datasets in the literature, expanding the algorithmic capacity of Mihm will work to simultaneously address the ongoing issue of data limitations and bias, especially given the numerous disease entities and high degree of patient diversity in dermatology. Lastly, future studies will report on the performance of Mihm in subtyping melanocytic lesions, ASL, and BCC, as well as the reliability of Mihm in determining margin status when applicable.

## Conclusions

There are challenges to be overcome at every phase of cancer management, making early detection, accurate classification, and timely treatment are paramount for improved patient outcomes. Our multi-level skin lesion detection system classifies 5 core lesion types, including melanocytic lesions, BCC, and ASL with exceptionally high sensitivity, surpassing that of other published AI models. Furthermore, Mihm not only delivers a slide-level classification, but it also aids pathologists in targeting specific diagnostic regions of WSIs and determines the distance between these regions and non-epidermal margins.

Until now, many AI models have fallen short of performing on par with or only slightly outperform dermatopathologists, likely owing to the absence of hand-drawn, pixel-level annotations in training sets, technical issues such as overfitting, and a lack of counter-training for continual improvement during model development. While we believe that AI has the potential to transform skin cancer diagnostic workflows and that algorithms that integrate both SL and SSL will shape the future of digital dermatopathology, this transformation should occur in conjunction with the expert dermatopathologists who implement these robust AI models in clinical practice and who will continue to contribute substantially to advancements in software, the quality and diversity of datasets, and user interfaces. When the performance characteristics of AI models exceed those of trained experts alone, and include features (i.e., localization, subtyping, and margin detection) that further expedite the course of patient care, AI algorithms not only help dermatopathologists to improve accuracy and time to diagnosis, but also save lives.

## Funding

Funding for this study was provided by Pathology Watch, Inc.

## Conflict of Interest

JR, TG, FB, AL are employees at Pathology Watch Inc, and are holders of equity and/or stock options in Pathology Watch Inc. RM, BB, DW, EG and GO are equity and/or stock option holders in Pathology Watch Inc. BB is a non-commercial consultant for Core Diagnostics (Menlo Park, CA). BB and GO are former consultants for Castle Biosciences.

## Appendix A Supplementary data

Supplementary material Image 1
