# Supplementary material for: High-fidelity detection, subtyping, and localization of five skin neoplasms using supervised and semi-supervised learning
Source: J Pathol Inform. 2022 Nov 26;14:100159. doi: 10.1016/j.jpi.2022.100159 (PMC9731861; doi:10.1016/j.jpi.2022.100159)
Supplement: Supplementary file 1 — Supplementary material [file mmc1.docx]

**Supplementary Material**

**Table S1**. Specimen source/site and clinical history keyword exclusion lists used to filter cases prior to running Mihm.

|  | **Specimen Source/Site** |
| --- | --- |
| **Keyword exclusion list** | mucosa, gingiva, tongue, palate, anus, anal, penis, penile, vulva, vulval, vagina, vaginal, scrotum, scrotal, nail, tissue, joint, conjunctival, conjunctiva, buccal, mucosal, gingival, palatal, labia |
|  |  |
|  | **Specimen Clinical History** |
| **Keyword exclusion list** | dermatitis, allergic, eczema, hypersensitivity, scabies, tinea, HSV, Zoster, herpes, SLE, lupus, eruption, rash, GA, sarcoid, alopecia, planus, condyloma, bullous, BP, pemphigus, psoriasis, nodosum, atopic, grovers, pleva, MF, dyshidrotic, pityriasis, urticaria, sweet, leukemia, faciale, nummular, darier, drug, necrobiosis, pyogenic, pyoderma, panniculitis, morphea, eczematous, urticarial, herpetic, pemph, pemphigal, CD, derm, contact, blister, lichen, vasculitits, sarcoidosis, granuloma, gram, onychomycosis, dermatophyte, sweets, telogen  HSV: herpes simplex virus, SLE: systemic lupus erythematosus, GA: granuloma annulare, BP: bullous pemphigoid, MF: mycosis fungoides, CD: contact dermatitis |

Table S2. “Other” skin conditions included in the supervised training set in the present study

| **Skin Conditions (OTHER)** |
| --- |
| Epidermal Inclusion Cyst |
| Dermatofibroma |
| Solar Lentigo |
| Lichenoid Keratosis |
| Inflammation |
| Excision Biopsy Site Changes |
| No Lesion |
| Hemangioma |
| Pilar Cyst |
| Angiolipoma |
| Neurofibroma |
| Lipoma |
| Angiofibroma |
| Keloid |
| Acrochordon |
| Prurigo Nodularis |
| Folliculitis |
| Pyogenic Granuloma |
| Sebaceous Gland Hyperplasia |
| Scar |
| Chondrodermatitis Nodularis Helicis |
| Psoriasis |
| Ulceration |
| Reactive Changes |
| Pilomatrixoma |
| Dilated Pore of Winer |
| Porokeratosis |
| Angioleioyoma |
| Cystically Dilated Follicle |
| Lichen Simplex Chronicus |
| Acantholytic Dyskeratosis |
| Granuloma Annulare |
| Molluscum |
| Lymphangioma |
| Excoriation |
| Hidrocystoma |
| Trichofolliculoma |
| Vitiligo |
| Palisaded Encapsulated Neuroma |
| Poroma |
| Inverted Follicular Keratosis |
| Dermoid Cyst |
| Angiokeratoma |
| Apocrine Cystadenoma |
| Acantholytic Acanthoma |
| Hidradenoma |
| Syringocystadenoma |
| Syringoma |
| Erosion |
| Xanthoma |
| Dermatophytosis |
| Atypical Fibroxanthoma |
| Xanthelasma |
| Follicular Cyst |
| Trichilemmoma |
| Onychodystrophy |
| Osteoma Cutis |
| Myxoid Cyst |
| Spiradenoma |
| Tattoo |
| Epithelioid Nerve Sheath Tumor |
| Sclerotic Fibroma |
| Milium Cyst |
| Alopecia |
| Steatocystoma |
| Warty Dyskeratoma |
| Spongiosis |
| Pilar Sheath Acanthoma |
| Acquired Digital Fibrokeratoma |
| Epidermolytic Acanthoma |
| Pseudoepitheliomatous Hyperplasia |
| Pilomatricoma |
| Cylindroma |
| Acrospiroma |
| Clear Cell Acanthoma |
| Folliculosebaceous Cystic Hamartoma |
| Psoriasiform Dermatitis |
| Syringocystadenoma Papilliferum |
| Paget's Disease |
| Grover's Disease |
| Poroid Hidradenoma |
| Tumor of the Follicular Infundibulum |
| Xanthogranuloma |
| Leiomyoma |
| Supernumerary Nipple |
| Desmoplastic Trichoepithelioma |
| Chondroid Syringoma |
| Condyloma |
| Sebaceous Adenoma |
| Rosacea |
| Telangiectasia |
| Solar Elastosis |
| Superficial Acral Fibromyxoma |
| Melanotic Macule |
| Intracorneal Hemorrhage |
| Fibrofolliculoma |
| Fibroepithelial Polyp |
| Solar Purpura |
| Fibrous Papule |

Table S3. Mimickers included in the Mihm validation dataset

| **DIAGNOSIS** | **MIMIC** |
| --- | --- |
| Acantholytic acanthoma | ASL |
| Acantholytic dyskeratosis | ASL |
| Acquired digital fibrokeratoma | VV |
| Acrospiroma | BCC |
| Angiokeratoma | VV |
| Chondrodermatitis nodularis helicis | ASL |
| Chondroid syringoma | BCC and ASL |
| Clear cell acanthoma | ASL |
| Cylindroma | BCC |
| Desmoplastic trichoepithelioma | BCC |
| Dilated pore of Winer | VV and ASL |
| Epidermal nevus | SK |
| Epidermolytic acanthoma | ASL and VV |
| Extramammary Paget's Disease | ASL and melanocytic |
| Focal acantholytic dyskeratosis | ASL |
| Folliculitis | ASL |
| Folliculosebaceous cystic hamartoma | BCC |
| Grover's Disease | ASL |
| HAK with spongiosis | BCC |
| Hidradenoma papilliferum | BCC |
| Hidradenoma/acrospiroma | BCC and ASL |
| Inverted follicular keratosis | ASL |
| Leiomyoma | ASL |
| Lentigo inflamed with melanophages | melanocytic |
| Lichenoid keratosis with melanophages | melanocytic |
| Molluscum | ASL and VV |
| Paget's Disease | ASL and melanocytic |
| Pilar sheath acanthoma | SK |
| Pilomatricoma | BCC |
| Poroid hidradenoma | BCC |
| Poroma | BCC |
| Proliferating pilomatricoma | BCC |
| Prurigo nodularis | VV |
| Pseudoepitheliomatous hyperplasia | ASL |
| Psoriasiform dermatitis | ASL, SK, VV |
| Pyogenic granuloma | ASL |
| SCC, keratoacanthoma type | VV |
| Sebaceous adenoma | BCC and ASL |
| SK, clonal type | ASL |
| Spiradenoma | BCC |
| Supernumerary nipple | SK |
| Syringocystadenoma papilliferum | BCC |
| Syringoma | BCC and ASL |
| Trichilemmoma | VV and ASL |
| Trichoepithelioma | BCC |
| Trichofolliculoma | BCC |
| Trichofolliculoma | ASL and SK |
| Tumor of the follicular infundibulum | ASL and SK |
| Warty dyskeratoma | VV |
